# Navigating second-line therapy after immunotherapy in advanced HCC☆^☆^

**DOI:** 10.1016/j.jhepr.2025.101630

**Published:** 2025-10-11

**Authors:** Arndt Vogel, Anna Saborowski, Lorenza Rimassa, Anthony El-Khoueiry

**Affiliations:** 1 Department of Gastroenterology, Hepatology, Infectious Diseases and Endocrinology, Hannover Medical School, Carl-Neuberg-Straße 1, 30625 Hannover, Germany; 2 UHN Division of Hepatology, Toronto General Hospital, Toronto, Canada; 3 Princess Margeret Cancer Center, Toronto, Canada; 4 Department of Biomedical Sciences, Humanitas University, Pieve Emanuele, Milan, Italy; 5 Medical Oncology and Hematology Unit, Humanitas Cancer Center, IRCCS Humanitas Research Hospital, Rozzano, Milan, Italy; 6 Division of Medical Oncology, University of Southern California, Norris Comprehensive Cancer Center, Keck Medicine of USC, 1441 Eastlake Ave, Los Angeles, CA, 90022, United States

**Keywords:** Hepatocellular carcinoma, Intra-arterial therapies, Tyrosine kinase inhibitor, Local regional therapies, Systemic therapy, Checkpoint inhibitor

## Abstract

In recent years, systemic treatment options for hepatocellular carcinoma have expanded significantly, with pivotal phase III trials reporting unprecedented improvements in survival and response outcomes. However, these advances have largely focused on first-line strategies, challenging existing treatment sequences and raising questions about the optimal therapeutic approach following disease progression. Several therapeutic options are available in second and later lines — including immunotherapies, tyrosine kinase inhibitors, and local therapies — but so far comprehensive head-to-head comparisons to establish the ideal treatment sequence are lacking. Consequently, most evidence guiding second-line therapy has been derived from *post hoc* analyses, real-world data, and select prospective studies, primarily based on phase II designs. In clinical practice, treatment decisions integrate this evolving evidence base with patient-specific factors such as prior treatment response, liver function, and performance status, often within the constraints of regulatory and accessibility considerations. Ongoing research is investigating novel approaches, including the continuation of immunotherapy following progression, the application of CAR T cells, and targeted treatments against specific markers like fibroblast growth factor 19 and glypican-3. This review summarises the current evidence for second-line and subsequent therapies, explores key considerations in treatment sequencing, and highlights emerging strategies that may further refine the therapeutic landscape for hepatocellular carcinoma.

## Graphical abstract

**Figure:**
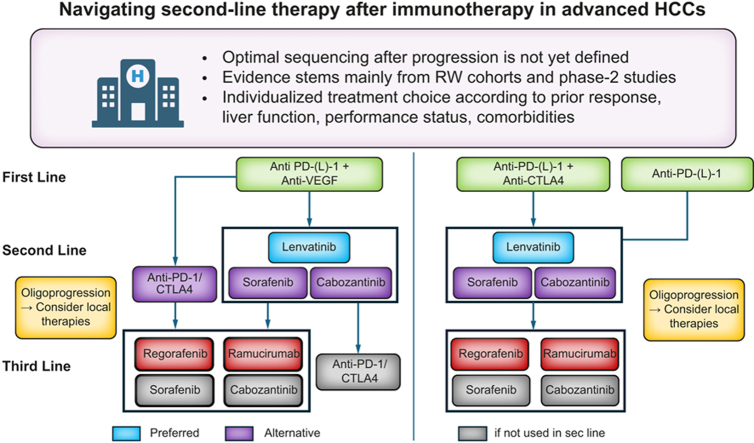

**Keypoints**

• With more than half of patients potentially eligible for second-line therapies after progression on first-line treatment, there is increasing clinical and research focus on optimal sequencing strategies, particularly following atezolizumab-bevacizumab.
• Tyrosine kinase inhibitors (*e.g.* sorafenib, lenvatinib, regorafenib, cabozantinib) remain essential in second-line therapy. Lenvatinib and sorafenib, though initially approved for first-line use, have demonstrated clinical benefit post-immune checkpoint inhibitor progression based on real-world data and small trials.
• In selected patients with oligo-progressive disease, integrating local ablative treatments (*e.g.* SBRT, TACE, SIRT) with continued systemic therapy may prolong disease control, although evidence remains limited and largely retrospective.
• Retreatment with immunotherapy – either by modifying ICI components (*e.g.* adding CTLA-4 blockade) or switching agents – is being actively investigated. Early evidence suggests potential benefit in a subset of patients, but efficacy remains unclear and should be pursued in trials.
• New agents are in development targeting Wnt/β-catenin, GPC-3, the FGF19-FGFR4 axis, and the tumour microenvironment. Some, like irpagratinib (FGFR4 inhibitor), and fostrox (liver-activated prodrug), have shown early promise in phase I/II studies.

## Introduction

**Fig. 1:**
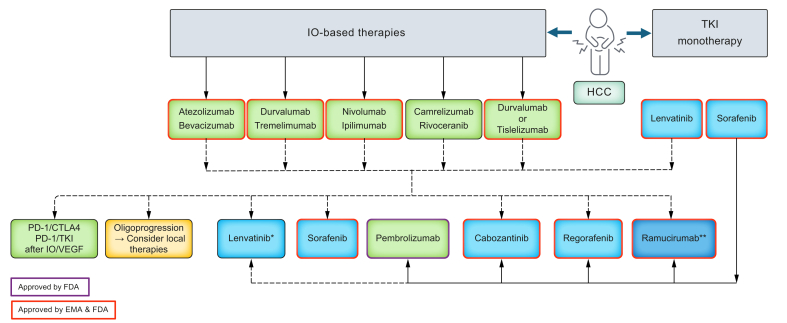
Summary of approved and non-approved treatment options for HCC in first and second lines. HCC, hepatocellular carcinoma; IO, immune-oncology; TKI, tyrosine kinase inhibitor. ∗ Not after lenvatinib 1^st^ line. ∗∗only in patients with AFP >400 mg/dl.

Hepatocellular carcinoma (HCC) is the most common primary liver cancer and often presents on a background of cirrhosis.[1], [2], [3], [4] The management of HCC necessitates a multidisciplinary approach, with treatment decisions influenced by an array of factors, including liver function, tumour-specific characteristics and the patient's performance status. The Barcelona Clinic Liver Cancer staging system is the most commonly employed framework for classifying HCC based on these factors, and it is endorsed by most Western clinical guidelines.[2], [3], [4] Treatment modalities for HCC are categorised into potentially curative options – such as liver transplantation, surgical resection or ablation – and palliative approaches, which include radiation, embolisation, and systemic therapy (Fig. 1).

Systemic therapy is the preferred treatment modality for patients with advanced-stage disease and in those with intermediate-stage disease who do not qualify for, or have progressed on, local therapies. Survival of patients treated with systemic agents has significantly improved over time and, with the approval of nine treatment regimens by the FDA and EMA, sequential therapy can now be routinely considered for patients with advanced HCC. The selection of systemic therapy for an individual patient will be influenced by several factors including efficacy and toxicity, and the presence of contraindications or predictive factors. In general patients should have Child-Pugh A liver disease and an Eastern Cooperative Oncology Group performance status (ECOG PS) of 0-1, consistent with the population in which the evidence base was generated. For most patients, combination therapy including a programmed death 1 (PD-1)/programmed death ligand 1 (PD-L1) inhibitor represents the first-line treatment of choice. According to data from the pivotal trials, approximately 50% to 60% of patients with advanced HCC are able to proceed to second-line treatment after failure of first-line strategies.[5], [6], [7], [8], [9], [10] This figure is anticipated to rise with advances in first-line therapies that improve overall prognosis and with the evolving adoption of systemic therapy in earlier stages of HCC. In real-world cohorts, which also include patients who do not meet the inclusion criteria of clinical trials, this number appears to be slightly lower, with roughly 30-50% of individuals who initially received atezolizumab plus bevacizumab as first-line treatment transitioning to second-line therapies.[11], [12], [13]

The systemic treatment landscape for advanced HCC has undergone significant evolution. Historically, first-line studies often compared novel agents to sorafenib and more recently to lenvatinib, while second-line studies used placebo as the comparator. This approach has resulted in a lack of direct efficacy comparisons between available drugs.^4^^,^^14^^,^^15^ To address this gap, network meta-analyses have been employed to integrate direct and indirect comparisons, providing a more comprehensive estimation of treatment efficacy. Meta-analyses have demonstrated that sequential multitargeted tyrosine kinase inhibitors (TKIs) in second line can significantly prolong survival, with a median overall survival (OS) of around 12 months and a median progression-free survival (PFS) of approximately 4 months.[16], [17], [18], [19]

Immune checkpoint inhibitor (ICI)-based combinations have now been established as the first-line standard of care; yet, no results from phase III second-line studies after progression on ICI-based therapies have been reported. IMbrave251 is one of the first studies exploring second-line options after failure of atezolizumab and bevacizumab in first line (NCT04770896). Currently available second-line therapies were evaluated and approved after progression on sorafenib but are now used after progression on ICI-based treatments with limited evidence. Real-world data are playing an increasingly vital role in shaping treatment guidance for second-line therapies. Observational studies and patient registries provide valuable data on treatment patterns and outcomes across diverse patient populations.

In clinical practice, several strategies guide the selection of second-line therapies. Switching to TKIs after immunotherapy failure is a common approach. However, there is limited evidence to definitively guide which TKI is superior in this setting. If sorafenib is chosen, well-established third-line options are available. There is increasing indirect evidence showing higher efficacy for lenvatinib compared to sorafenib, but subsequent treatment options after lenvatinib failure lack robust evidence.^9^ Cabozantinib has shown promise in a phase II study with patients previously treated with ICIs.^20^^,^^21^ Rechallenge with immunotherapy after failure of first-line immune checkpoint inhibitors is currently being explored in clinical trials.^22^ Locoregional therapy (LRT), including stereotactic body radiation therapy (SBRT), can also be integrated into second-line strategies, particularly for patients with oligo-progressive disease.^23^^,^^24^

Second-line treatment for HCC is an increasingly complex and individualised decision-making process. Management strategies must account for liver function, performance status, prior treatment responses and toxicities, and the specific patterns of disease progression. TKIs, ICIs (including rechallenge approaches), and LRT have a role in this setting. Ongoing clinical trials and the accumulation of real-world data are crucial to further refine treatment algorithms and optimise outcomes for patients with advanced HCC. In the following review, we provide a more detailed overview of potential second-line therapies and treatment strategies.

## Rationale and data to support TKIs in second line and beyond

TKIs are orally administered small molecules that inhibit multiple pathways involved in angiogenesis, tumour growth, and metastasis Sorafenib inhibits RAF-1, BRAF, vascular endothelial growth factor receptor (VEGFR)1–3, and platelet-derived growth factor receptor (PDGFR)-β, kinases involved in angiogenesis and tumour proliferation.^25^ In the phase III SHARP trial, sorafenib as first-line therapy significantly improved median OS (10.7 *vs.* 7.9 months) and TTP (5.5 *vs.* 2.8 months) compared to placebo. Grade 3 treatment-related adverse events (TRAEs) were more frequent with sorafenib and included diarrhoea, hand-foot skin reaction, and weight loss. Discontinuation rates due to AEs and the incidence of serious AEs were similar in the two arms. These findings were confirmed by the Asia-Pacific phase III trial, which enrolled 226 patients randomised 2:1 to sorafenib or placebo.^26^

Lenvatinib, an oral TKI targeting VEGFR1–3, FGFR1–4, PDGFR-α, RET, and KIT, and inhibiting the pro-neoangiogenic and immunosuppressive effects of the tumour microenvironment, was tested in the open-label phase III REFLECT trial and shown to be non-inferior to sorafenib in terms of OS (13.6 *vs.* 12.3 months, hazard ratio 0.92).^27^ Lenvatinib demonstrated superior PFS (7.3 *vs.* 3.6 months), TTP (7.4 *vs.* 3.7 months), and objective response rate (ORR) (18.8% *vs.* 6.5%) compared to sorafenib. Serious TRAEs and AEs leading to treatment discontinuation were more frequent in the lenvatinib arm. Recent phase III trials offer additional insights into the efficacy of lenvatinib and sorafenib within the evolving therapeutic landscape. Specifically, phase III trials using lenvatinib as the control arm in first-line therapy for HCC include LEAP-002 (pembrolizumab and lenvatinib *vs*. lenvatinib) and Checkmate 9DW (nivolumab and ipilimumab *vs*. lenvatinib or sorafenib, with lenvatinib being used in 85% of patients in the control arm).^9^^,^^10^ In both studies, lenvatinib resulted in a median PFS of 8-9 months and median OS of 19-20 months. Similarly, the sorafenib arms of the COSMIC-312, CheckMate 459, the non-VP4 arm of IMbrave150, and the 4-year survival data from the HIMALAYA trial show remarkably consistent Kaplan-Meier curves,[5], [6], [7]^,^[28], [29], [30], [31] collectively establishing a median OS of approximately 14 to 15 months as a benchmark for sorafenib in the current treatment landscape.

According to most national and international guidelines, sorafenib and lenvatinib can be considered as a subsequent treatment option after failure of ICI-based first-line therapies for advanced HCC.^1^^,^^3^^,^^4^ Both drugs have been shown to be effective options after atezolizumab-bevacizumab according to real-world data and a small single-arm phase II study (Table 2).[32], [33], [34]

**Table 2:** Phase I/II trials in second line after ICI-based first-line therapies.

Study	South Korea Hong Kong^20^	Global^22^		South Korea^35^	South Korea^81^	USA^55^
n	74	79 (AB)	28	50	40	19
Drug	Cabo	Rego + Pembo		Lenva	Rego	Bot/Bal
**Baseline characteristics**						
ECOG PS 0	68%	47%	56%	18%	60%	53%
ECOG PS 1	32%	53%	44%	82%	40%	47%
BCLC A	0%	0%	0%	12%	0%	NA
BCLC B	6%	19%	30%	12%	2.5%	NA
BCLC C	94%	81%	70%	76%	97.5%	NA
EHS	NA	66%	56%	NA	NA	68%
MVI	NA	32%	19%	24%	25%	NA
CP A	100%	100%	100%	100%	100%	NA
ALBI 1	NA	NA	NA	NA	42.5%	53%
ALBI 2	NA	NA	NA	NA	57.5%	47%
AFP >400 ng/ml	NA	NA	NA	44%	40%	53%

**Efficacy data**						
mOS	9.9	NR	NR	8.6	10.5	12.3
PFS	4.1	2.8	4.2	5.4	3.5	4.4
HR (95% CI)	NA	NA	NA	NA	NA	
PR	6%	6%	11%	12%	10%	17%
DCR	83%	54%	74%	84%	82.%%	72%

**Safety**						
Grade ≥3 AEs	Low platelets 6% Hypertension 4% Fatigue 4%	HFSR 8% Asthenia 6% Hypertension 3%		Hypertension 8% Proteinuria 6% AST elevation 6%	Low platelets 5%	imAEs 37% Diarrhoea/colitis 16% Hepatitis 16%
Subsequent therapies	27%	NA	NA	NA	NA	NA

AEs, adverse events; AFP, alpha-fetoprotein; ALBI, albumin-bilirubin; AST, aspartate aminotransferase; BCLC, Barcelona Clinic Liver Cancer; CP, Child-Pugh; DCR, disease control rate; EHS, extrahepatic spread; ECOG PS, Eastern Cooperative Oncology Group performance status; HFSR, hand foot skin reaction; HR, hazard ratio; imAEs, immune-mediated adverse events; mOS, median overall survival; MVI, macrovascular invasion; PFS, progression-free survival; PR, partial response.

Lenvatinib was assessed in a Korean multicentre phase II study as a second-line treatment in 50 patients previously treated with atezolizumab-bevacizumab, with ECOG PS 0-1 and Child-Pugh class A.^35^ The primary endpoint was PFS, and patients received lenvatinib at the standard dose of 12 mg/day if ≥60 kg or 8 mg/day if <60 kg. Median PFS was 5.4 months (95% CI 5.3-5.6), and median OS was 8.6 months (95% CI 8.1-NA), although OS data were immature and require longer follow-up. TTP on prior atezolizumab-bevacizumab treatment did not affect PFS on lenvatinib. Partial response (PR) and stable disease (SD) were reported in 12% and 72% of patients, for a disease control rate (DCR) of 84%. TRAEs were consistent with prior studies, with no new safety signals. The most common grade 3-4 TRAEs were hypertension (8%), anorexia, proteinuria, and aspartate aminotransferase elevation (6% each).

A meta-analysis of seven studies with 387 patients treated with sorafenib or lenvatinib after progression on ICI-based therapy showed a pooled ORR of 26% and a DCR of 63% with sorafenib and lenvatinib.^19^ The pooled OS was 11.45 months and PFS was 3.78 months. While lenvatinib was associated with slightly longer median OS (12.42 months) and median PFS (5.15 months) compared to sorafenib (10.75 and 2.58 months, respectively), the difference did not reach statistical significance. The AE profiles of sorafenib and lenvatinib after atezolizumab-bevacizumab treatment were similar to what had previously been reported for both agents in pivotal first-line trials. Common all-grade AEs included hand-foot syndrome (43%) and fatigue (41%).

Regorafenib, cabozantinib and ramurciumab were initially evaluated in patients previously treated with sorafenib (Table 1).[36], [37], [38] Regorafenib blocks the activity of multiple protein kinases involved in angiogenesis, proliferation, tumour microenvironment, and metastasis, including VEGFR1-3, TIE2, KIT, RET, RAF-1, BRAF, PDGFR, and FGFR.^39^ Its efficacy was initially demonstrated in patients who tolerated sorafenib at doses of ≥400 mg daily, and this benefit was later confirmed regardless of prior sorafenib tolerance.^40^ In the double-blind phase III RESORCE trial, regorafenib achieved longer median OS compared to placebo (10.6 *vs.* 7.8 months) and ORR by RECIST 1.1 was 7%. Grade 3-4 TRAEs occurred in 50% of patients (46% grade 3, and 4% grade 4), and 10% discontinued therapy due to TRAEs.^36^ Cabozantinib demonstrates immunomodulatory activity and targets multiple receptor tyrosine kinases involved in tumour pathogenesis, including the pro-angiogenic growth factor receptors VEGFR and MET, as well as the TAM family of kinases (TYRO3, AXL, MER), which contribute to immunosuppression in the tumour microenvironment.^29^ In the double-blind phase III CELESTIAL trial, cabozantinib improved OS compared to placebo, with a median OS of 10.2 *vs.* 8.0 months in the whole cohort and 11.3 *vs.* 7.2 months in second line. The CELESTIAL trial was unique in allowing up to two prior lines of therapy. ORR by RECIST 1.1 was 4%. Grade 3-4 AEs occurred in 68% of patients, and 16% discontinued therapy due to TRAEs.^37^

**Table 1:** Phase III trials in second line.

Study	RESORCE^36^		CELESTIAL^37^		REACH-2^42^	
n	379/194		470/237		197/197	
Drug	Rego	Placebo	Cabo	Placebo	Ram	Placebo
**Baseline characteristics**						
ECOG PS 0	65%	67%	52%	55%	57%	58%
ECOG PS 1	35%	33%	48%	45%	43%	42%
BCLC A	1%	0%	0%	0%	0%	0%
BCLC B	14%	11%	9%	10%	17%	21%
BCLC C	86%	89%	91%	90%	83%	97%
EHS	70%	76%	79%	77%	72%	74%
MVI	29%	28%	27%	34%	36%	35%
CP A	98%	97%	99%	99%	100%	100%
ALBI 1	43%	42%	39%	43%	43%	44%
ALBI 2	56%	58%	61%	57%	57%	56%
AFP >400 ng/ml	43%	45%	41%	43%	100%	100%

**Efficacy data**						
mOS	10.6	7.8	10.2	8.0	8.1	5.3
PFS	3.1	1.5	5.2	1.9	2.8	1.5
HR (95% CI)	OS: 0.63 (0.50-0.79) PFS: 0.46 (0.37-0.56)		OS: 0.76 (0.63-0.92) PFS: 0.44 (0.36-0.52)		OS: 0.71 (0.53-0.95) PFS: 0.45 (0.34-0.60)	
PR	7%	3%	4%	<1%	5%	1%
DCR	65%	36%	64%	33%	60%	39%

**Safety**						
Grade ≥3 AEs	Hypertension 16% HFSR 13% Bilirubin increase 11%		HFSR 17% Hypertension 17% Diarrhoea 17%		Liver failure 18.3% Hypertension 12.7% Bleeding 5.1%	
Subsequent therapies	20%	28%	25%	30%	26.9%	28.4%

AEs, adverse events; AFP, alpha-fetoprotein; ALBI, albumin-bilirubin; BCLC, Barcelona Clinic Liver Cancer; CP, Child-Pugh; DCR, disease control rate; EHS, extrahepatic spread; ECOG PS, Eastern Cooperative Oncology Group performance status; HFSR, hand foot skin reaction; HR, hazard ratio; mOS, median overall survival; MVI, macrovascular invasion; PFS, progression-free survival; PR, partial response.

Ramucirumab, a recombinant monoclonal antibody targeting VEGFR-2, was the first non-TKI administered intravenously to be evaluated in advanced HCC. In the phase III REACH trial, ramucirumab did not significantly improve OS in patients previously treated with sorafenib.^41^ However, a predefined subgroup analysis indicated a potential OS benefit in patients with baseline alpha-fetoprotein (AFP) levels ≥400 ng/ml – a finding subsequently confirmed in the REACH-2 trial, which exclusively enrolled patients meeting this AFP criterion.^42^ In addition, a pooled meta-analysis of prospectively collected quality-of-life data demonstrated a statistically significant advantage of ramucirumab over placebo. In a small expansion cohort of the REACH-2 study, the activity of ramucirumab was shown in patients following non-sorafenib systemic therapies.^43^ Although ramucirumab is an approved treatment option for patients with elevated AFP levels, it should not be viewed as the default therapy for this subgroup. Other agents, particularly TKIs, have demonstrated efficacy irrespective of AFP status and may offer broader therapeutic benefit. The benefit of ramucirumab following ICI and anti-VEGF combinations in first line has not been evaluated, and it is unknown whether prior exposure to VEGF-targeted agents may influence the efficacy or resistance profile of ramucirumab.

Regorafenib, as shown in the prospective observational REFINE study^40^ and cabozantinib, as shown in a *post hoc* analysis of the CELESTIAL trial, have shown efficacy and safety after ICIs, even when administered beyond second-line therapy. More recently, prospective single-arm phase II trials of cabozantinib^20^ and regorafenib^44^ in patients who progressed on ICIs provide insight into their activity and safety in this setting. Cabozantinib was tested in an Asian multicentre phase II study in patients with ECOG PS 0-1, Child-Pugh class A, and up to two prior lines of systemic therapy.^20^ Forty-seven patients were enrolled: 27 and 20 patients had received one and two prior therapies, respectively. Median follow-up was 11.2 months. Median PFS was 4.1 months (95% CI 3.3-5.3), and median OS was 9.9 months (95% CI 7.3-14.4), with a 1-year OS rate of 45.3%. PR and SD were reported in 6.4% and 76.6% of patients, respectively, for a DCR of 83%. Among 21 patients who had received prior atezolizumab-bevacizumab, median PFS and OS were 4.3 and 11.8 months, respectively. Among 17 patients who had received second-line cabozantinib following an active first-line regimen, median PFS and OS were 4.3 (95% CI 3.3-11.0) and 14.3 (95% CI 9.0-NR) months, respectively. The number of prior therapies was identified as an independent prognostic factor (one *vs.* two, hazard ratio 0.37, 95% CI 0.15-0.90, *p* = 0.03). TRAEs were consistent with prior studies, with no new safety signals. The most common grade 3-4 TRAEs were thrombocytopenia (6.4%) and hypertension (4.3%). Thirty-five patients (74.5%) had a dose reduction and the median dose of cabozantinib was 40 mg daily. Six patients (12.8%) discontinued treatment due to AEs.

Regorafenib was evaluated in a Korean multicentre phase II study as a second-line treatment in patients previously treated with atezolizumab-bevacizumab with ECOG PS 0-1 and Child-Pugh class A (Table 2).^44^ The primary endpoint was PFS, and patients received regorafenib at the standard dose of 160 mg daily for the first 3 weeks of each 4-week cycle. Forty patients were enrolled and median follow-up was 6.5 months. Median PFS was 3.5 months (95% CI 3.0-4.0), and median OS was 10.5 months (95% CI 8.3-11.1), with a 6-month OS rate of 65%. OS was statistically significantly shorter in patients with the shortest TTP (<2.3 months) on prior atezolizumab-bevacizumab (*p* <0.001), while no statistically significant difference was shown for PFS. PR and SD were reported in 10% and 72.5% of patients, respectively, for a DCR of 82.5%. TRAEs were consistent with prior studies, with no new safety signals, and the most common grade 3-4 TRAE was thrombocytopenia (5%).

Based on the available data, either shifting to sorafenib or lenvatinib, originally approved in the first-line setting, or adopting regorafenib or cabozantinib, approved as further-line options after sorafenib, are acceptable strategies recognised by most of the international guidelines. A simulation model built on the data from pivotal phase III trials has been used to identify the best treatment strategy.^33^ Lenvatinib and sorafenib emerged as the most effective second-line options, and the sorafenib-cabozantinib sequence following atezolizumab-bevacizumab achieved a median OS of 28 months in the simulation model.^33^

## Rationale and data to integrate local therapies for patients with oligo-progression

In the treatment landscape of advanced cancers, the phenomenon of oligometastatic progression – where a limited number of metastases progress while most disease sites remain controlled under systemic therapy – poses unique therapeutic challenges and opportunities; this scenario may allow for a strategic combination of local ablative therapy (LRT) with continued systemic management. This integrated approach often involves continuing with the current regimen of ICIs or transitioning to TKIs or alternative ICIs, depending on the clinical response and tumour characteristics. LRTs including radiofrequency ablation, intra-arterial therapies (such as transarterial chemoembolisation and selective intra-arterial radiotherapy), and SBRT, may be employed in this context. These modalities not only target the oligo-progressive sites but also potentially enhance the tumour’s immunogenicity. LRT-induced cell death can lead to the release of tumour-associated antigens and the activation of antigen-presenting cells, thereby potentially re-sensitising the tumour microenvironment to immunotherapy.

Emerging evidence underscores the benefit of incorporating LRT in conjunction with systemic immunotherapy in HCC. A case series documented the outcomes of LRT in managing oligo-progression in five patients with advanced HCC, including cases with extrahepatic metastases or major portal vein tumour thrombosis.^45^ These patients underwent SBRT to intrahepatic oligo-progressive sites while maintaining immunotherapy, achieving a median OS of approximately 24 months – a notable improvement over historical cohorts. Furthermore, Sindhu *et al.* analysed 30 patients with solid tumours, including 16 HCC cases, who underwent SBRT following oligo-progression during ICI therapy.^24^ This group exhibited a median PFS of 7.1 months, with a two-year OS rate of 82.8%. Importantly, there were no instances of grade ≥3 acute toxicities, and all patients continued ICI therapy for at least 4 months post-SBRT. Additionally, a retrospective study from Taiwan indicated that approximately 30% of patients with HCC, initially responsive to immunotherapy but later showing oligo-progression, were suitable for LRT. Those who received LRT after subsequent progression displayed prolonged OS.^23^ Patients treated with LRT for oligo-progression had a significantly longer median OS compared to those who did not (48.4 *vs.* 20.5 months, *p* <0.001). In contrast, the median OS for patients with polymetastatic progression receiving LRT did not differ significantly from those not receiving LRT (27.7 *vs.* 25.5 months, *p* = 0.794).

These findings advocate for a nuanced approach to cancer management in the oligometastatic setting, leveraging the synergistic potential of LRT and systemic therapies to optimise patient outcomes. This approach not only controls localised disease progression but also potentially reinvigorates systemic immune responses against tumours, illustrating a promising avenue for extending survival and maintaining quality of life in patients with advanced cancers. While the emerging concept of treating oligo-progressive disease may offer a rational approach to prolong disease control in selected patients, it is important to emphasise that the current evidence supporting this strategy remains limited. Most data are derived from small retrospective studies or early-phase trials, and there is a lack of high-quality prospective evidence. Consequently, existing guideline recommendations remain weak, and this approach should be considered investigational and applied with caution in clinical practice. Overall, this remains an area worthy of further investigation in prospective trials.

## Immunotherapeutic approaches after progression on first-line checkpoint inhibitor-based therapy

As detailed above, treatment with TKIs represents a common approach once patients have progressed on or become intolerant to ICI therapy. In parallel, there is scientific rationale and clinical interest in approaches that can potentially continue to harness anti-cancer immunity after checkpoint inhibitor therapy. One such approach revolves around the continuation of anti-PD-1 or PD-L1 therapy in second line with the addition of an immuno-modulatory agent not used in first-line treatment. The first evaluation of this approach was in a phase II study of regorafenib and pembrolizumab in patients who progressed on ICI-based therapy in first line. Regorafenib, a multi-kinase inhibitor, has been shown to promote anti-cancer immunity through the induction of M1 macrophage polarisation and enhancement of CD8+ T-cell proliferation and activation.^46^ The combination of regorafenib and anti-PD-1 therapy is superior to either agent alone in preclinical models and has achieved an ORR of 31% and a median OS of 24.9 months in the first-line treatment of HCC.^47^ The second-line trial of regorafenib and pembrolizumab enrolled patients who had progressed on one prior line of therapy containing an anti-PD(L)1 agent (Table 2);^22^ cohort 1 (n = 68) was limited to patients who had received first-line atezolizumab and bevacizumab and cohort 2 (n = 27) enrolled patients who had received other ICI-based therapy including single-agent anti-PD-1 or anti-PD-L1 antibodies. The ORR by RECIST 1.1 was 5.9% and the median PFS was 2.8 months (95% CI 2.4–3.9) in cohort 1. In cohort 2, the ORR was 11.1% and median PFS was 4.2 months (95% CI 2.9–6.8). Grade 3 and-4 TRAEs occurred in 37% and 3% of patients, respectively, with the most common being asthenia, hand-foot skin reaction and hypertension. TRAEs leading to discontinuation of regorafenib or both regorafenib and pembrolizumab occurred in 11% and 3% of patients, respectively. The rate of immune-mediated events was 22%. Overall, this trial demonstrated only modest activity, with a response rate and PFS comparable to those achieved with single-agent regorafenib, suggesting that no synergistic effects were achieved. This is reminiscent of data in the first-line setting, wherein the combination of immunotherapy and lenvatinib (LEAP-002) or cabozantinib (COSMIC-312)^9^^,^^28^^,^^29^ did not demonstrate strong synergistic activity. A similar approach investigating the continuation of anti-PD-L1 therapy after progression on first-line ICIs is being evaluated in the randomised phase III IMbrave251 trial, which is comparing atezolizumab plus lenvatinib or sorafenib *vs.* sorafenib or lenvatinib in patients who progressed on atezolizumab and bevacizumab (NCT04770896).

Another strategy is to (co-)target inhibitory immune checkpoints other than PD-(L)1, such as CTLA-4. CTLA-4 inhibits activated T cells and regulatory T cells (Tregs) by binding to its ligands B7-1 and B7-2 on antigen-presenting cells. In addition to counteracting this inhibitory effect, CTLA-4 antibodies enhance anti-cancer immunity by depleting Tregs.^48^ Based on the HIMALAYA phase III trial, a single dose of anti-CTLA-4 (tremelimumab) and regular interval administration of anti-PD-L1 (durvalumab) has been approved by the EMA and FDA in first-line HCC, and more recently, dual checkpoint inhibition with nivolumab and ipilimumab also successfully entered frontline therapy.^7^^,^^8^^,^^10^ Prior to being tested in first line, nivolumab and ipilimumab showed remarkable activity in patients with advanced HCC who were previously treated with sorafenib in the phase I/II CheckMate 040 trial. Patients were randomised 1:1:1 to either nivolumab 1 mg/kg plus ipilimumab 3 mg/kg every 3 weeks (4 doses), followed by nivolumab 240 mg every 2 weeks (arm A); nivolumab 3 mg/kg plus ipilimumab 1 mg/kg every 3 weeks (4 doses), followed by nivolumab 240 mg every 2 weeks (arm B); or nivolumab 3 mg/kg every 2 weeks plus ipilimumab 1 mg/kg every 6 weeks (arm C). Although the response rate was comparable across the three arms, arm A, which received the higher dose of ipilimumab, demonstrated the most notable results, with an ORR of 34% and a median OS of 22.2 months (34/50; 95% CI 9.4–54.8 months).^49^^,^^50^ While the combination of nivolumab and ipilimumab received US FDA accelerated approval based on these results, its role and activity after progression on first-line ICI therapy are not fully established. Several small retrospective series suggest that the combination has activity in patients previously treated with ICI-based therapy, either as single agents or in combination with an anti-VEGF antibody or a TKI. The limitations of these series include the small patient numbers, the variety of ICI treatments given in first line, the known biases related to patient selection, and the lack of rigor in determining true progression in first line. Further, many of the series have only been reported in abstract form. Nonetheless, ORRs ranging between 13% and 22% have been noted across these reports. One of the largest studies identified 58 patients out of 994 ICI-treated individuals who were rechallenged with an ICI in second or later lines of therapy. Interestingly, the response rate was comparable to that observed in the first-line setting and included responses in patients who had not responded to prior ICI treatment. The authors concluded that ICI rechallenge was safe and provided clinical benefit in a meaningful subset of patients with HCC.^51^ Wong *et al.* reported on 25 patients, 96% of whom had received single-agent anti-PD-1 therapy in first line and 60% of whom received nivolumab and ipilimumab in second line, while the rest received the combination in the third and fourth lines;^52^ in this series, the ORR and SD rate were 16% and 24%, respectively, with a median OS of 10.9 months (95% CI 3.99 to 17.8). There were no new safety signals, with one patient having grade 3 hepatitis, one having grade 3 colitis, and 12% requiring systemic immunosuppressants. Another series reported on 32 patients, 81% of whom had received anti-PD-(L)-1 plus anti-VEGF (50% atezolizumab and bevacizumab and 31% other anti-PD-(L)-1 plus anti-VEGF), and 69% of whom had received two or more prior lines of treatment. The ORR and SD rate were 22% and 25%, respectively, with a median OS of 9.2 months (95% CI 5.9-not reached). Immune-related AEs occurred in 41% of patients with 19% being grade 3 or 4; there was one grade 5 event related to immune hepatitis.^53^ A prospective phase II study of nivolumab and ipilimumab in patients who progressed on first-line atezolizumab and bevacizumab is ongoing (NCT05199285). Further, novel or enhanced CTLA-4 antibodies are under evaluation for advanced HCC. One of the lead substances is botensilimab, a multifunctional CTLA-4 inhibitor with mutations in the Fc region that increase binding to Fc gamma receptors on antigen-presenting cells and natural killer (NK) cells. This results in enhanced priming, expansion and memory T cell formation, activation of antigen-presenting cells and myeloid cells, and Treg depletion.^54^ A single-arm prospective expansion cohort evaluated botensilimab in combination with balstilimab, a PD-1 antibody, in 19 patients with advanced HCC who had progressed on first-line ICI-based therapy. Patients had received a median of two prior lines of therapy (range 1–7); all had received prior PD-(L)1 as part of combination therapy (58% atezolizumab/bevacizumab) and 63% had received prior TKIs. The ORR by RECIST 1.1 was 17% (3 PRs/18; 95% CI 3%–40%). The clinical benefit rate (complete response, PR or SD ≥18 weeks) was 47% (95% CI 24%–71%). Median OS was 12.3 months (95% CI 8.4–21.4). Grade 3 immune-mediated TRAEs occurred in 37% of patients, with diarrhoea, hepatitis and adverse skin reactions being the most common.^55^ In summary, checkpoint inhibitor therapy in the second-line setting and beyond remains under investigation in patients previously treated with first-line ICI therapy. There is currently no definitive evidence that it improves survival in this setting. Specifically, the continuation of anti-PD-(L)-1 therapy in second line while switching the second agent (for example, switching the second agent from an anti-VEGF antibody to a TKI) should only be done in the context of a clinical trial. There is an emerging body of evidence to suggest potential activity of anti-CTLA-4 antibody therapy in combination with anti-PD-(L)-1 in second line and beyond in patients who received immune checkpoint-based therapy in first line that did not target CTLA-4.

## Novel drug development in second line and beyond

Agents of various therapeutic classes targeting tumour genomics or the tumour microenvironment are under development for advanced HCC after progression on first-line therapy. While an exhaustive review of this area is beyond the scope of this manuscript, below we highlight selected examples of novel therapeutics currently in early-phase clinical trials.

### Targeting the Wnt/β-catenin pathway

The role of WNT/β-catenin signalling in HCC pathogenesis is supported by the high mutational rate in *CTNNB1*, which encodes β-catenin. Activation of the WNT/β-catenin pathway may also occur through other mechanisms, including mutations in related genes and pathways, such as *AXIN* and *APC*. Besides its interaction with multiple oncogenic pathways, the Wnt/β-catenin pathway has been associated with immune exclusion, albeit *post hoc* analysis from pivotal trials failed to find an association between β-catenin mutational status and response to immunotherapy.^56^^,^^57^ Several compounds that inhibit or modulate the Wnt/β-catenin pathway are currently under early clinical investigation, with results pending. For example, E7386 is a specific inhibitor of the interaction between CBP and β-catenin and acts as a downstream Wnt/β catenin pathway modulator.^58^ In a dose escalation study of E7386 in combination with lenvatinib, a PR rate of 24% was noted;^59^ an ongoing phase Ib study is assessing the combination of E7386 and lenvatinib *vs*. lenvatinib alone in patients with HCC who received one prior line of an immunotherapy-based regimen.^60^ Another agent that targets this pathway is ALN-BCAT, a chemically modified small-interfering RNA encapsulated in a lipid nanoparticle formulation, which generates robust and highly specific reductions of *CTNNB1* mRNA.^61^ A first-in-human trial of ALN-BCAT in patients with HCC with a Wnt pathway activating mutation is currently ongoing (NCT06600321).

### Targeting glypican-3

Glypican-3 (GPC-3) serves as a critical therapeutic target in HCC due to its selective overexpression in tumour cells compared to normal adult liver tissues.^62^ This unique expression profile underpins its potential both as a biomarker for early disease detection and a target for therapeutic intervention. The involvement of GPC-3 in key oncogenic pathways, such as Wnt, YAP, and Hedgehog, suggests that it plays a role in tumour growth and metastasis, making it a promising candidate for targeted therapy.^63^

Therapeutically, a range of strategies targeting GPC-3 are currently under evaluation in clinical trials,^64^ including monoclonal antibodies, bi-specific antibodies, peptide vaccines, chimeric antigen receptor (CAR) T-cell therapies, and CAR NK cell therapies.[65], [66], [67] For instance, a phase I clinical study of C-CAR031, a GPC3-specific CAR T-cell therapy, demonstrated promising anti-tumour activity in patients with advanced HCC.^68^ Peptide vaccines targeting GPC-3 have shown the ability to induce cytotoxic T lymphocytes and improve prognosis in some patients with HCC.^69^ Preclinical studies have also highlighted the potential of GPC-3-specific CAR NK cells for HCC treatment.^70^ Combination therapies involving GPC-3 targeting and other immunotherapeutic agents are also being explored to enhance treatment efficacy.

### Targeting fibroblast growth factor 19

Fibroblast growth factor 19 (FGF19) and its receptor, FGFR4, present a significant axis in cancer biology, particularly in HCC. FGFR4 is part of the FGFR family of receptor tyrosine kinases, which includes FGFR1 through FGFR4. In the context of HCC, FGF19, the ligand for FGFR4, is frequently amplified and overexpressed, leading to the activation of the FGFR4 signalling pathway and contributing to tumour development and progression.^71^ The aberrations in the FGF19-FGFR4 pathway are not limited to overexpression; gene mutations within *FGFR4* have also been implicated in tumorigenesis across various cancer types. FGF19 overexpression has been reported in approximately 30% of patients with HCC.^72^^,^^73^ The frequent activation and alteration of this pathway in HCC highlight its potential as a therapeutic target, offering opportunities for the development of targeted therapies that could disrupt this axis and potentially halt or reverse tumour growth. Fisogatinib (BLU-554) is a selective FGFR4 inhibitor that demonstrated activity in early clinical trials for FGF19-driven HCC. Despite encouraging response rates, the drug was not further developed due to adverse effects and the rapid emergence of secondary resistance.^74^ More recently, irpagratinib, a novel second-generation highly selective inhibitor of FGFR4 has shown promise alone and in combination with atezolizumab in heavily pre-treated patients with HCC.^75^^,^^76^

### Chemotherapy

Systemic chemotherapy failed to show clinical benefit in Caucasian populations, while high-dose intra-arterial chemotherapy is often used in mostly Asian populations, suggesting a role for liver-directed chemotherapy.^77^ Recently, a novel approach that exploits the first pass metabolism of a prodrug to enhance liver exposure has been introduced into clinical trials: after oral administration, fostrox is metabolised to troxacitabine, which acts as a nucleoside analogue. The drug is currently being investigated alone and in combination with lenvatinib in the second-line setting, aiming to exploit anti-angiogenic synergy and complementary activity against extrahepatic lesions.^78^^,^^79^ A phase Ib/IIa study, including 21 patients with HCC, recently reported an ORR of 24% with TTP of 10.9 months, respectively. The combination will be further investigated in second line in a randomised phase II design, with lenvatinib plus placebo as the comparator.^80^

## Conclusion

The efficacy of second-line therapies in patients with HCC with preserved performance status and liver function has been clearly established, but therapeutic choices have been guided by phase III data following progression on first-line sorafenib. With the advent of immunotherapy as the new first-line standard of care, the cards have been reshuffled, and while it is commonly agreed upon that all eligible patients should be offered further lines of therapy, it remains a matter of debate what the "optimal" sequential treatment strategy should be. Evidence supporting the efficacy of available therapies beyond first-line ICI-based therapy remains limited to single-arm phase II trials and real-world data. Further, the ongoing lack of predictive biomarkers creates a significant gap in treatment selection. As a result, clinical investigation is, at this time, focused on conceptual strategies, aiming to identify and prioritise approaches that may offer greater benefits, particularly for an all-comer patient population. In this scenario, ICIs and TKIs continue to be the key players. Thus, current second-line treatment strategies largely revolve around critical decisions: whether to continue immunotherapy beyond progression, whether to transition to a TKI, modify immunotherapy in further lines, or explore combinations of immunotherapy with TKIs. A deeper understanding of the resistance mechanisms and the tumour microenvironment in patients who have progressed on ICI-based therapy will be critical to guide rational drug development in the future. Lastly, efforts to leverage biomarker-tailored approaches that may lead to enhanced patient selection continue to be needed in this space.

## Abbreviations

AE, adverse event; AFP, alpha-fetoprotein; CAR, chimeric antigen receptor; CTLA-4, cytotoxic T-lymphocyte–associated protein 4; DCR, disease control rate; ECOG PS, Eastern Cooperative Oncology Group performance status; FGF19, fibroblast growth factor 19; FGFR, FGF receptor; GPC-3, glypican-3; HCC, hepatocellular carcinoma; ICI, immune checkpoint inhibitor; LRT, locoregional therapy; NK, natural killer; ORR, objective response rate; OS, overall survival; PD-1, programmed death 1; PDGFR, platelet-derived growth factor receptor; PD-L1, programmed death ligand 1; PFS, progression-free survival; PR, partial response; SBRT, stereotactic body radiation therapy; SD, stable disease; TKI, tyrosine kinase inhibitor; TRAE, treatment-related adverse event; Treg, regulatory T cell; TTP, time to progression; VEGFR, vascular endothelial growth factor receptor.

## Financial support

The authors did not receive any financial support to produce this manuscript.

## Authors’ contributions

All authors contributed equally to the literature review and search, formatting, writing, and editing of the manuscript and figures. All authors replied to the peer reviewers’ comments and approved the submitted version.

## Conflict of interest

AV reports personal fees from Roche, Bayer, BMS, Lilly, EISAI, AstraZeneca, Ipsen, MSD, Sirtex, BTG, Servier, Zymeworks Terumo, Imaging Equipment Ltd (AAA). AS reports personal fees from Roche, Servier, BMS. LR reports consulting fees from AbbVie, AstraZeneca, Basilea, Bayer, BMS, Eisai, Elevar Therapeutics, Exelixis, Genenta, Hengrui, Incyte, Ipsen, Jazz Pharmaceuticals, MSD, Nerviano Medical Sciences, Roche, Servier, Taiho Oncology, Zymeworks; lecture fees from AstraZeneca, Bayer, BMS, Eisai, Guerbet, Incyte, Ipsen, Roche, Servier; travel expenses from AstraZeneca and Servier; research grants (to Institution) from AbbVie, AstraZeneca, BeiGene, Exelixis, Fibrogen, Incyte, Ipsen, Jazz Pharmaceuticals, MSD, Nerviano Medical Sciences, Roche, Servier, Taiho Oncology, TransThera Sciences, Zymeworks. AEK reports consulting fees from BMS, Roche/Genentech, Exelixis, Astrazeneca, Eisai, Merck, Abbvie, Jansen, Elevar, Terumo, Servier, Qurient, Senti Biosciences; research grants (to institution) from Astex, Astrazeneca, Fulgent, Auransa.

Please refer to the accompanying ICMJE disclosure forms for further details.
